# Development of chitosan-folate modified PLGA nanoparticles for targeted delivery of diosgenin as an anticancer agent

**DOI:** 10.1007/s12672-024-00957-7

**Published:** 2024-04-03

**Authors:** Fatemeh Teymouri, Ehsan Karimi

**Affiliations:** Department of Biology, Mashhad Branch, Islamic Azad University, Mashhad, Iran

**Keywords:** Chitosan, Diosgenin, Drug delivery, Folate, PLGA nanoparticle

## Abstract

Diosgenin as a potential phytoconstituent and steroidal saponin manifested significant anticancer agents against various cancers. To enhance its solubility and bioavailability in cancer treatment, we loaded diosgenin (PubChem CID: 99474) in poly(lactic-co-glycolide) (PLGA) nanoparticle coated with folic acid-chitosan (Da-PFC-NPs). The diosgenin nano-formulation was characterized and its antioxidant and anticancer properties were surveyed respectively. The obtained results illustrated that the Da-PFC-NPs were spherical and stable with a size of 218 nm and a polydispersity index of 0.41. The Da-PFC-NPs indicated potential free radical scavenging using ABTS and DPPH assay. Meanwhile, it demonstrated selective toxicity against the TUBO breast cancer cell with IC_50_ values of 104.45 μg/ml and did not show toxicity on normal cells (I929 cell line). The *invivo* funding exhibited that Da-PFC-NPs notably  altered the liver enzymes (AST, ALT, ALP) and immunoglobulins (IgA, IgG, IgM). Besides that, different doses of Da-PFC-NPs (50 and 100 mg/kg) remarkedly enhance the expression of caspase 3 and decrease HER2 genes. In light of this experiment, we can conclude that Da-PFC-NPs have promise as novel carrier for improving the delivery of diosgenin in cancer therapy.

## Introduction

Plant-based pharmaceuticals were the pedestal of traditional medicine and they continue as key players in modern pharmacotherapy, particularly the treatment of cancer and infectious diseases [1]. The high toxicity and adverse effects of common chemotherapeutic medicines, even at therapeutic doses, have encouraged both pharmacists and patients to focus more on phytomedicine. Since, in addition to the lower adverse effects and cost-effectiveness, phytochemicals have shown great anticancer action at different stages of tumor growth [2, 3]. However, despite the phytochemicals' excellent promise as anticancer agents, there are some limitations regarding their pharmacokinetics. Phytochemicals have poor solubility which reduces the permeability and therefore leads to the low bioavailability of the compound. Moreover, given that they have unstable biological structure, improper molecular size, easy absorption by healthy tissue, and are subjected to gastric or enzymatic breakdown, early drug loss and fast clearance happen [3, 4].

With advances that nanotechnology has brought to medicine, drug delivery systems have been promoted to nanosized systems with diverse surface-engineering methodologies for targeted drug administration. Among these delivery technologies, phytomedicine-based nano vehicles are regarded as one of the most biocompatible, efficient, and effective anticancer drug delivery systems [3]. Nanocarriers enhance solubility, bioavailability, pharmacological activity, stability, while reducing the toxicity. Drug delivery via nanocarriers also fulfills the need for site-specific drug administration and accumulation, controlled release, and big molecule delivery [3, 4]. To achieve these goals in nano drug delivery, the integrity of nanoparticles (NPs) in the bloodstream must be maintained which is one of the main biological challenges in controlled delivery.

Poly (lactic-co-glycolic acid), PLGA, is a FDA approved biodegradable copolymer with great potential as a nanocarrier since it degrades to non-toxic byproducts (H_2_O and CO_2_) and allows a controlled sustainable drug release. PLGA nanoparticles can be modified in order to minimize the off-target chance. Conjugation with receptors (such as antibodies, epidermal factors, and peptides), PEGylation, and binding with ligands (like DSPE and folic acid (FA)), or combination with other polymers like chitosan (CS) improves the specificity of cell targeting. Chitosan is another biocompatible and nontoxic material for drug encapsulation which enhances the nanoparticle maintenance in blood stream by adding mucoadhesive properties to the delivery system. Moreover, both PLGA and cellular membranes are negatively charged, therefore, adding the positively charged chitosan can improve the system by enhancing the cellular uptake and internalization of the nanoparticle [3, 5, 6].

Diosgenin, a steroidal sapogenin, is a bioactive phytochemical that was classically used as a precursor in the synthesis of steroidal drugs. However, recent studies have discovered its therapeutic effect in the treatment of different diseases, including neurological and metabolic complications, infections, and cancers [7, 8]. Due to its anti-inflammatory, antioxidant, immunoregulatory, antiproliferative, and cytotoxic properties, diosgenin has shown a chemo preventive effect on different cancer cell lines, including squamous carcinoma, lung cancer, colon carcinoma, breast cancer, liver cancer and hepatocellular carcinoma [7, 9]. Considering the urgent call for substitution of conventional toxic chemical drugs with new cost-effective nontoxic therapeutics in cancer therapy and given the extensive pharmacological potential of diosgenin, it is time to take action on optimizing the administration to commercialize such natural bioactive compounds. Previous researchers have succeeded to nano capsulate diosgenin in couple of nanocarriers including noisome, silver, and iron oxide carriers [9, 10]. This study investigates to establish a diosgenin-loaded PLGA nanoparticle coated with folic acid-chitosan (Da-PFC-NPs) and to explore its antioxidant, and anticancer activities in vivo and in vitro system.

## Material and method

### Materials

Diosgenin (≥ 93%, Cas No: 512-04-9), poly lactic-co-glycolic acid 50/50 (PLGA), LMW chitosan (1526.5 *g*/mol), polyvinyl alcohol (PVA), folic acid (FA), 1-Ethyl-3-(3-dimethyl aminopropyl)carbodiimide (EDC) and N-Hydroxysuccinimide (NHS) were purchased from Sigma-Aldrich. Dichloromethane (DCM), 3- (4, 5-dimethylthiazol-2-yl)-2,5-diphenyltetrazolium bromide (MTT), and dimethylsulfoxide (DMSO) were purchased from Merck company. The 1.1-diphenyl-2-picrylhydrazyl (DPPH), Butylated hydroxyanisole (BHA) and acetonitrile HPLC grade were obtained from Fisher Scientific, UK. All requirements of cell culture were purchased from Invitrogen Company. Cell lines were purchased from the Cell Bank of Ferdowsi University of Mashhad, Iran.

### Synthesis of FA-CS

Folic acid was first dissolved in DMSO, and then EDC and NHS were added to the FA-containing solution. The resulting mixture got thorouly mixed for 1 h by stirrer in the dark and was filtered afterward. For binding of FA to CS, the CS solution was made by dissolving CS in 1% acetic acid at pH 4.7 and stirring. Finally, FA-NHS was applied dropwise to CS and incubated for 24 h on the stirrer to achieve FA-CS conjugation. After the incubation, the pH was adjusted to 9, and the precipitate (FA-CS) was recovered using a centrifuge. The precipitate was lyophilized after dialysis [11].

### Preparation of diosgenin loaded PLGA

The single emulsion solvent evaporation technique W/O was applied to load diosgenin to PLGA NPs. For this purpose, PLGA was dissolved in DCM. The organic phase solution was then supplemented with diosgenin and varying amounts of PVA were added to the prepared solution in two steps. Then, 4 ml of PVA (2%) was added and emulsified by a sonicator (4 min). The following step was adding 10 ml of PVA (0.1 percent) and incubating for 2 h on the stirrer. After the solvent had completely evaporated, the sample was centrifuged for 20 min at 13,000 rpm, and the supernatant was gathered to evaluate the efficacy of the diosgenin encapsulation [11].

### Surface modification of diosgenin-loaded PLGA- NPs with FA-CS

PLGA-diosgenin precipitate dissolved in DW and FA-CS precipitate dissolved in 1% acetic acid. Then, CS-FA solution was added dropwise to the PLGA-drug mixture. The resulting solution was stirred for 2 h, then centrifuged for 13 min at 13,000 rpm, and finally dried by a freeze dryer [11].

### Characterization of Da-PFC-NPs

The efficacy of the nanoparticle synthesis was explored via Dynamic light scattering (DLS), zeta potential (ZP), and scanning electron microscope (SEM). Samples were prepared for analysis by dissolving 1mg of Da-PFC-NPs in 10 ml of deionized distilled water (DW) at 25 ℃. The size and dispersion index, and the surface charge of the Da-PFC-NPs were evaluated by DLS and a Zetasizer Nano ZS90 (Malvern Instruments, Worcestershire, UK), respectively.

SEM was also used for exploring the morphology of synthetized nanoparticles. Sample preparation carried out by dissolving 1 mg of NPs in 10 ml of DW, and the sample were applied to aluminum foil dropwise and dried at room temperature. Then, the samples were coated with gold and inspected under a microscope [12].

### Evaluation of the encapsulation efficiency of folic acid and diosgenin

In order to confirm and find the extent of folic acid/diosgenin binding and the encapsulation, a high performance liquid chromatography (C18 column of 250 mm × 4.6 mm, temperature: 24 ± 1 ℃, mobile phase: ammonium acetate in distilled water/acetonitrile at 40:60 (v/v)) was performed as previously reported [13]. Free folate was applied as the standard. The area below the peak of the test sample was calculated and compared to the standard to evaluate the amount of folic acid in the test solution. A method similar to FA binding was used to evaluate the encapsulation rate of diosgenin.

### Antioxidant activity

Both ABTS and DPPH radical scavenging ability of Da-PFC-NPs were explored according to the previous reports [14, 15]. All tests were carried out in triplicates.

#### ABTS cation scavenging

Briefly, ABTS was dissolved in water to a concentration of 7 mM. For the ABTS + stock solution preparation, ABTS radical cation (ABTS +) was added to 2.45 mM K_2_S_2_O_8_, mixed together and incubated at room temperature overnight. Then, the ABTS + stock solution was diluted. The ABTS solution (1 ml) was combined with 1 ml of varied NPs concentrations and left at room temperature in the dark for 1 h. Eventually, absorbance at 734 nm was measured. The BHA was applied as the reference standard.

#### DPPH scavenging activity

The radical scavenging activity of Da-PFC-NPs was also investigated by colorimetric assay using DPPH. For this purpose, DPPH radicals were initially prepared by combining 1 mg of DPPH with 17 ml of 96% ethanol. Various concentrations of Da-PFC-NPs were prepared through serial dilution in 1.5 ml microtubes. Subsequently, 500 μl of DPPH free radicals were added to each microtube. The mixture was incubated at 37 ℃ for 30 min and the optical density at 517 nm was measured and recorded. The inhibition percentage of DPPH free radicals was calculated as follows: Percent (%) inhibition of DPPH = (A0 − A1∕A0) × 100%

### Cell culture and in vitro cytotoxicity assay

The cytotoxicity of Da-PFC-NPs was evaluated on normal (L929 cells), colon cancer (CT-26 cells), and Tubo (breast cancer) cell lines via MTT assay. Briefly, Cells were transferred to 96‐well culture plates at a density of 5 × 10^3^ cells/ml. Plates were incubated for 24 h and got treated with different concentrations of NPs afterward. A negative control with only culture media was also tested. After 48 h, the plates were incubated for 4 h with the MTT solution. After discarding the medium, 20 μl of DMSO was added to the wells. Eventually, the absorbance was measured by plate reader at a test wavelength of 570 nm. The negative control group was marked as 100 percent, and the findings were presented as a percentage of the negative control [12].

### Breast cancer mouse model

Male Balb/C mice (6–8 weeks) were obtained from the BuAli research center of Mashhad, Iran. Mice were housed on a 12:12-h light–dark cycle under controlled temperature and humidity, receiving water and food ad libitum. Mice got divided to the following four groups (n = 5): healthy control, tumoral group, NP50, and NP100 mg/kg/BW. To induce breast tumor development in mice, 100 μl of Tubo cell suspension were injected subcutaneously. As soon as the tumor appeared, mice in treatment groups were treated with Da-PFC-NPs at dose of 50 and 100 mg/kg/BW. The drug has been administered intraperitoneally daily for 28 days. Size of tumor was measured every 2 days in each test group during the treatment using a digital caliper. At the end of the treatment, mice were sacrificed and their blood and tumor tissue were collected for cellular and molecular investigation. All methods and procedures were carried out in accordance with the relevant guidelines and regulations. All methods are reported in accordance with ARRIVE guidelines.

### Tumor histopathology

Collected tumor tissue was rinsed by NaCl (0.9%) serum twice, fixed in 10% formalin, and got embedded in paraffin. The paraffinized samples were then cut into 5 μm thin layers. The prepared samples were stained with hematoxylin and eosin (H&E) according to the Elmore et al. [16]. The stained slides were analyzed by light microscopy.

### RNA isolation and RT-PCR

Total RNA was isolated from collected tissues according to the manufacturer’s protocol using Noragen kit. cDNA was synthesized from isolated mRNA using Quantitect Reverse Transcription kit (Qiagen, Hilden, Germany). The quantitative real-time PCR analysis was carried out on a Biorad-CFX96 Real Time PCR device following the standard procedure. Relative expression of caspase3 (Cas3) and human epidermal growth factor receptor 2 (HER2) genes were assessed in tumor tissue. Expression level of target genes was compared to GAPDH as the reference gene. The primer pairs’ sequences are addressed in Table 1.

**Table 1:** qPCR primer sequences

Genes	Forward (5ʹ- -3ʹ)	Reverse (5ʹ- -3ʹ)	Reference
Caspase 3	ctggactgtggcattgagac	acaaagcgactggatgaacc	[17]
HER2	cctcctaaaggacctagaggaaggc	caaggccaggagaggcactggggag	[18]
GAPDH	ccggatcgaccactacctgggcaac	gttccccacgtactggcccaggacca	[17]

### Blood markers of hepatic function and inflammation

Blood sample (1.5 ml) obtained by cardiac puncture and transferred into blood collection tubes. The tubes were centrifuged (3000 *g*, 15 min) and serum got collected. The liver function profile was evaluated by measuring alanine aminotransferase (ALT), aspartate aminotransferase (AST), and alkaline phosphatase (ALP) levels in serum samples. To investigate the mice's immune system response to the treatment, antibody titer (IgG, IgA, IgM) was also assessed.

### Statistical analysis

Data were analyzed using SPSS software (version 26) and presented as means ± SEM. Assessing the differences between control and test groups, we applied a one-way analysis of variance (ANOVA) followed by the LSD multiple comparisons test. The P value of less than 0.05 was considered statistically significant.

## Results and discusion

### Physicochemical analysis of Da-PFC-NPs

**Fig. 1:**
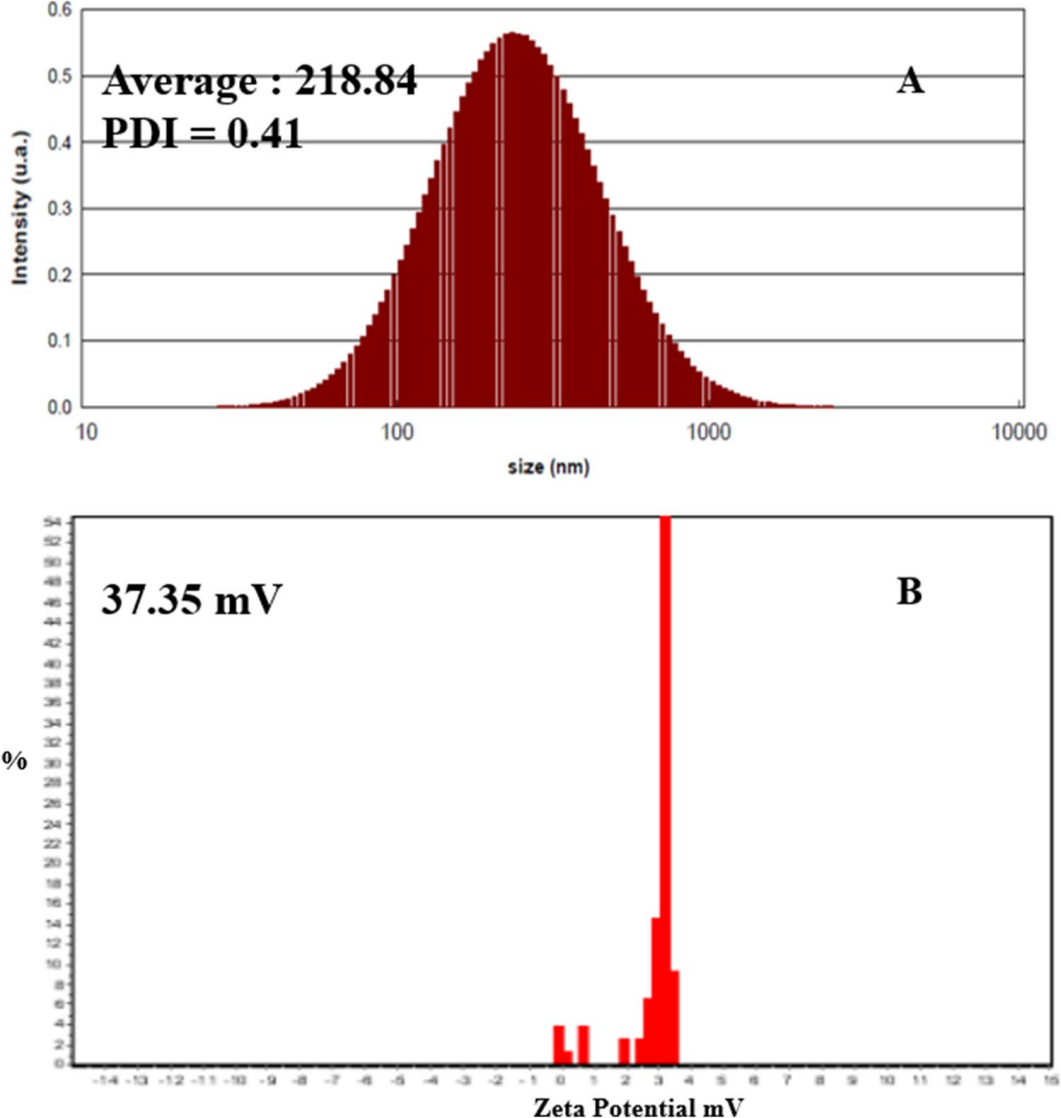
The DLS and Zeta potential analysis of Da-PFC-NPs

**Fig. 2:**
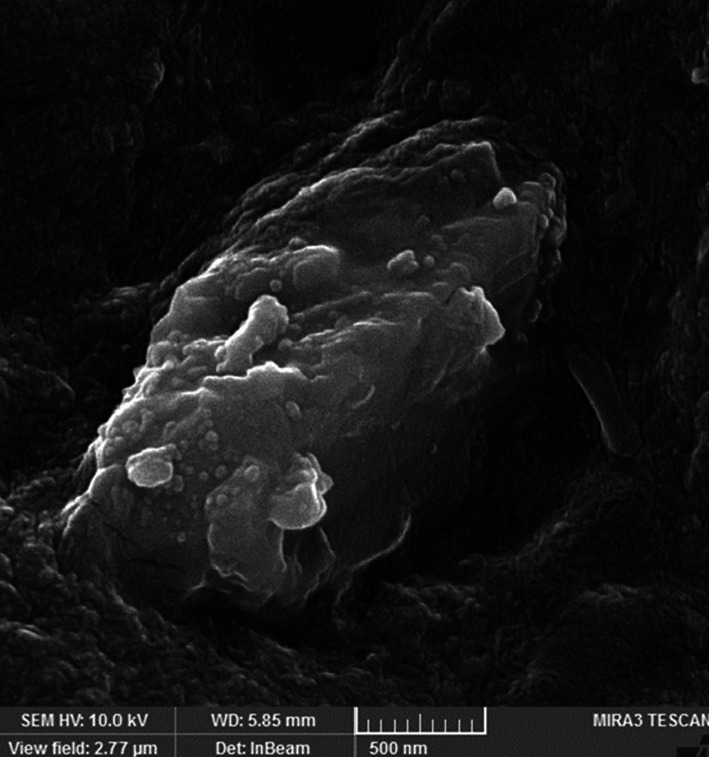
Field Emission Scanning Electron Microscope image of Da-PFC-NPs

According to previous experiments the nanoparticles size and polydispersity index (PDI) are the important factors that have an impact on the bioactive compounds releasing and solubility from the nanoparticle formulation. As presented in Fig. 1, the Da-PFC-NPs have an average size of 218 nm and surface charge of 37.35 mV that confirmed the stable nanoparticle for biological investigation. The FESEM images indicated spherical morphology that confirmed the Zetasizer nano analysis (Fig. 2). The diosgenin encapsulation and folic acid binding percentages in the nanoparticle complex were 94% and 65% respectively.

### Antioxidant capacity analysis of Da-PFC-NPs

**Fig. 3:**
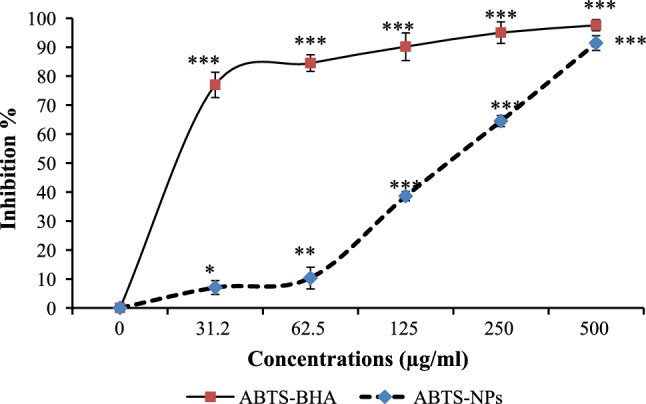
ABTS free radical scavenging activities of Da-PFC-NPs. The BHA were used as control. (N = 3, *P < 0.05, **P < 0.01 and ***P < 0.001)

**Fig. 4:**
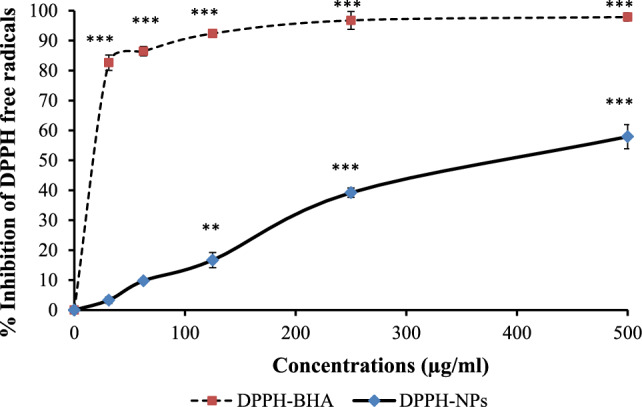
DPPH free radical scavenging activities of Da-PFC-NPs. The BHA were used as control. (N = 3, *P < 0.05, **P < 0.01 and ***P < 0.001)

The Da-PFC-NPs was significantly inhibited the ABTS^.+^ which was decreased to ABTS'. Percentage free radical scavenging at concentration of 250 μg/ml for nanoparticle and BHA as positive control were 64.46% and 95.02% respectively (Fig. 3). Similar to ABTS results, Da-PFC-NPs indicated potential DPPH-free radicals’ scavenger, since at concentration of 500 μg/ml the activity was 57.91% that was lower than reference antioxidant (Fig. 4). This data is an agreement with study that carried out by Sadeghzadeh et al. [19] that synthesized the PLGA nanoparticle modified with chitosan/folate for delivery of colchicine (COL-PPCF-NPs) and evaluated its antioxidant properties. They results illustrated that COL-PPCF-NPs significantly inhibited the free radicals of ABTS and DPPH with respective IC_50_ values of 108.07 and 361.61 mg/ml.

### Cytotoxic effects of Da-PFC-NPs

**Fig. 5:**
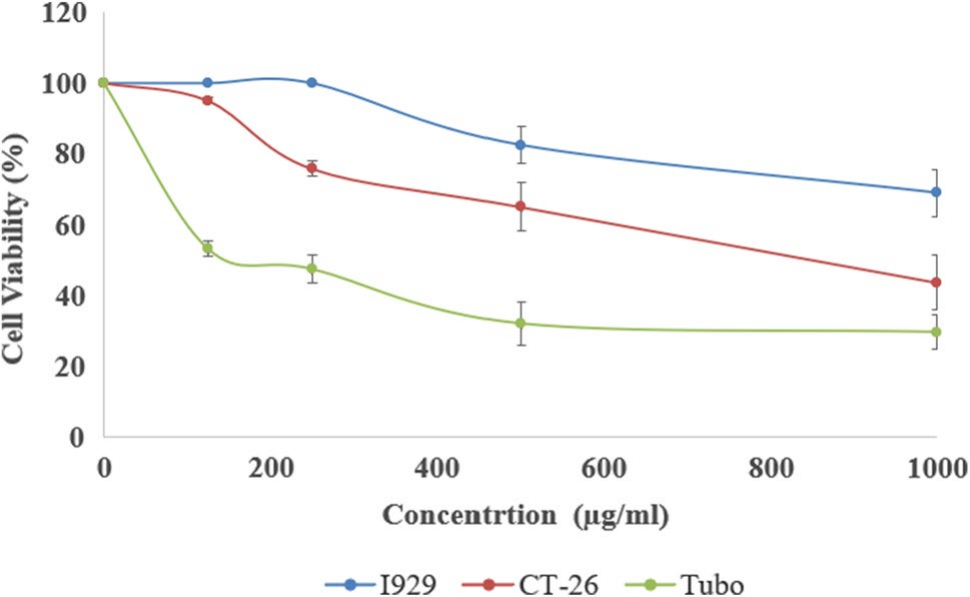
The anticancer potential of Da-PFC-NPs against Ct-26 and Tubo cancer cell line in comparison with I929 as a normal cell line

The analysis of antiproliferative properties of Da-PFC-NPs against the normal (L929 cells) and cancer cell lines (CT-26 and Tubo) is demonstrated in Fig. 5. Enhancement in Da-PFC-NPs concentration up to 1000 µg/ml diminish the cell viability of all cancer cells significantly (P < 0.001) in a dose depend manner. But, the Da-PFC-NPs exhibit low toxic impact on normal cell. The overall results manifested that Da-PFC-NPs showed strongest anticancer potential toward breast cancer cells (Tubo) compared to colon cancer (CT-26) with respective IC_50_ of 194.77 and 850.11 µg/ml. Therefore, the further experiment was carried out on this cell line. Similar results related to anticancer potential are indicated by Rahmati et al. [13]. They showed the strong cytotoxic effect of α-terpineol-PLGA nanoparticles coated with folic acid-chitosan against HT-29, PC3, AGS and MCF-7 cancer cells with a median concentration of 430, 657.8, 601.6 and 601.5 µg/ml and no toxicity were observed against normal cell (HFF cell line).

### Anti-tumour activity of Da-PFC-NPs

#### Blood analysis

Table 2 illustrates the blood parameters alteration like liver enzymes (AST, ALT, ALP) and immunoglobulins (IgA, IgG, IgM). In the TUBO tumor-bearing mice administrated with normal food (T2) the liver enzyme levels were significantly (P < 0.05) increased and immunoglobulins factors were decreased. Following the treatment with 50 and 100 mg/kg of Da-PFC-NPs were improved remarkably these parameters (P < 0.05).

**Table 2:** The blood analysis in the liver tissue of mice receiving different treatments

Treatment	AST(U/L)	ALT (U/L)	ALP (U/L)	IgA (mg/dl)	IgG (mg/dl)	IgM (mg/dl)
T1	167^b^	238^b^	280^bc^	6.4^b^	9.9^a^	6.8^b^
T2	192^a^	252^a^	310^a^	5.1^c^	7.5^bc^	6.3^b^
T3	127^d^	250^a^	307^a^	6.6^b^	7.8^bc^	7.2^a^
T4	152^c^	227^c^	285^b^	8^a^	8.9^b^	7.4^a^

T1: Control group, T2: TUBO tumor-bearing mice receiving normal food, T3: TUBO tumor-bearing mice receiving 50 mg/kg/BW of Da-PFC-NPs, T4: TUBO tumor-bearing mice receiving 100 mg/kg/BW of Da-PFC-NPs. Different letters (a, b, c, d) in the same column represent significant difference (P < 0.05). The analyses were performed in triplicates

### Tumor size, weigh and histopathology evaluation

**Fig. 6:**
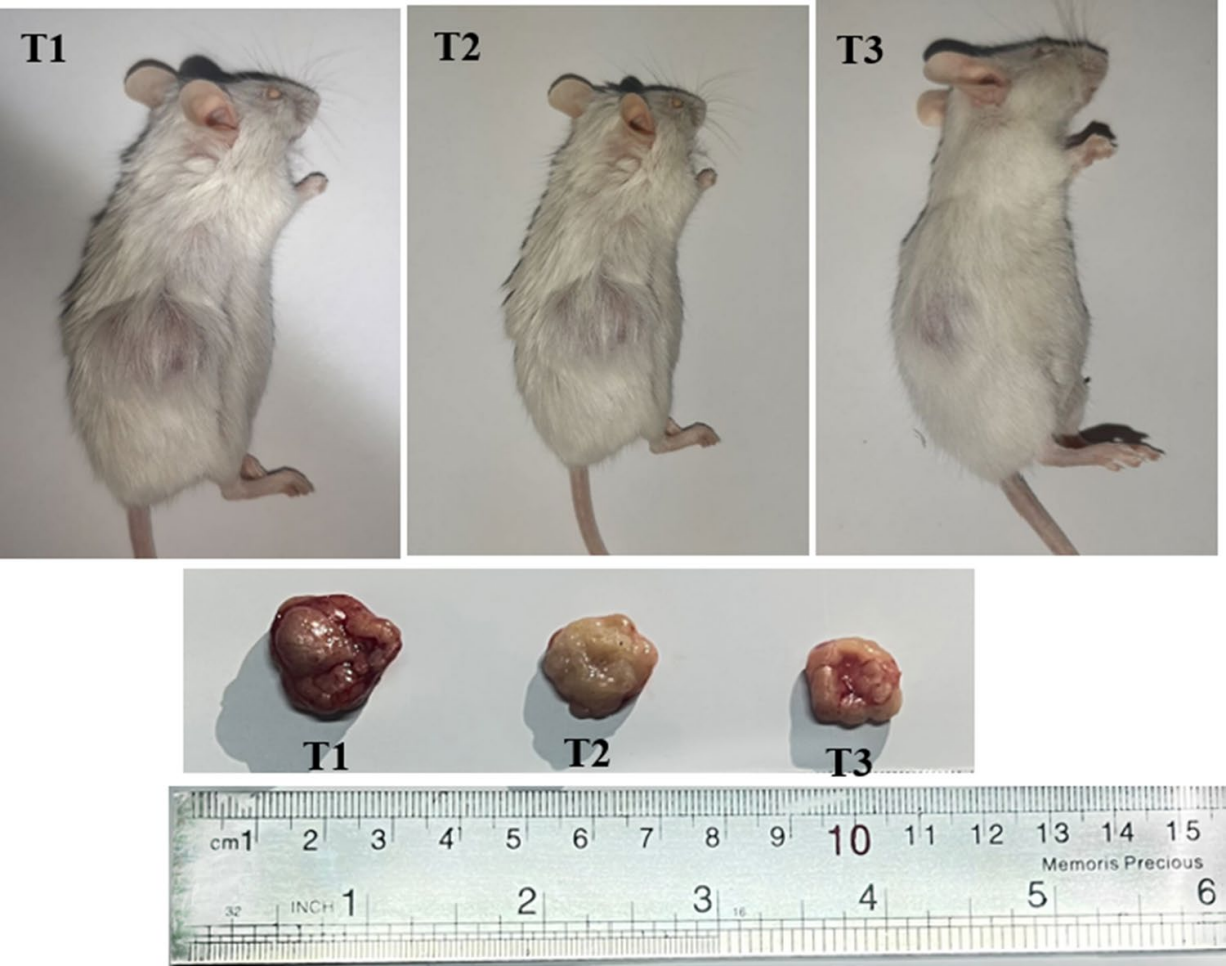
Comparison of the tumor size in TUBO tumor-bearing mice during experiments receiving different treatments. T1: TUBO tumor-bearing mice receiving normal food, T2: TUBO tumor-bearing mice receiving 50 mg/kg/BW of Da-PFC-NPs, T3: TUBO tumor-bearing mice receiving 100 mg/kg/BW of Da-PFC-NPs

Base on the results listed in Table 3 and Fig. 6, a significant difference was exhibited in the mean of tumor weight and size in TUBO tumor-bearing mice receiving different treatments. The mice group administrated with 50 and 100 mg/kg indicated more antiproliferative effect as compared to control. However, the impact of 100 mg/kg of Da-PFC-NPs was more prominent.

**Table 3:** The weight and size of tumor upon 28 days in TUBO tumor-bearing mice during experiment receiving different treatments

Tumor	T1	T2	T3
Weight (g)	2.6 ± 0.27a	1.9 ± 0.32b	1.5 ± 0.20c
Size (mm)	19.5 ± 5.42a	17.1 ± 6.92b	15.7 ± 7.36c

T1: TUBO tumor-bearing mice receiving normal food, T2: TUBO tumor-bearing mice receiving 50 mg/kg/BW of Da-PFC-NPs, T3: TUBO tumor-bearing mice receiving 100 mg/kg/BW of Da-PFC-NPs. Different letters (a, b, c, d) in the same row represent significant difference (P < 0.05). The analyses were performed in triplicates

**Fig. 7:**
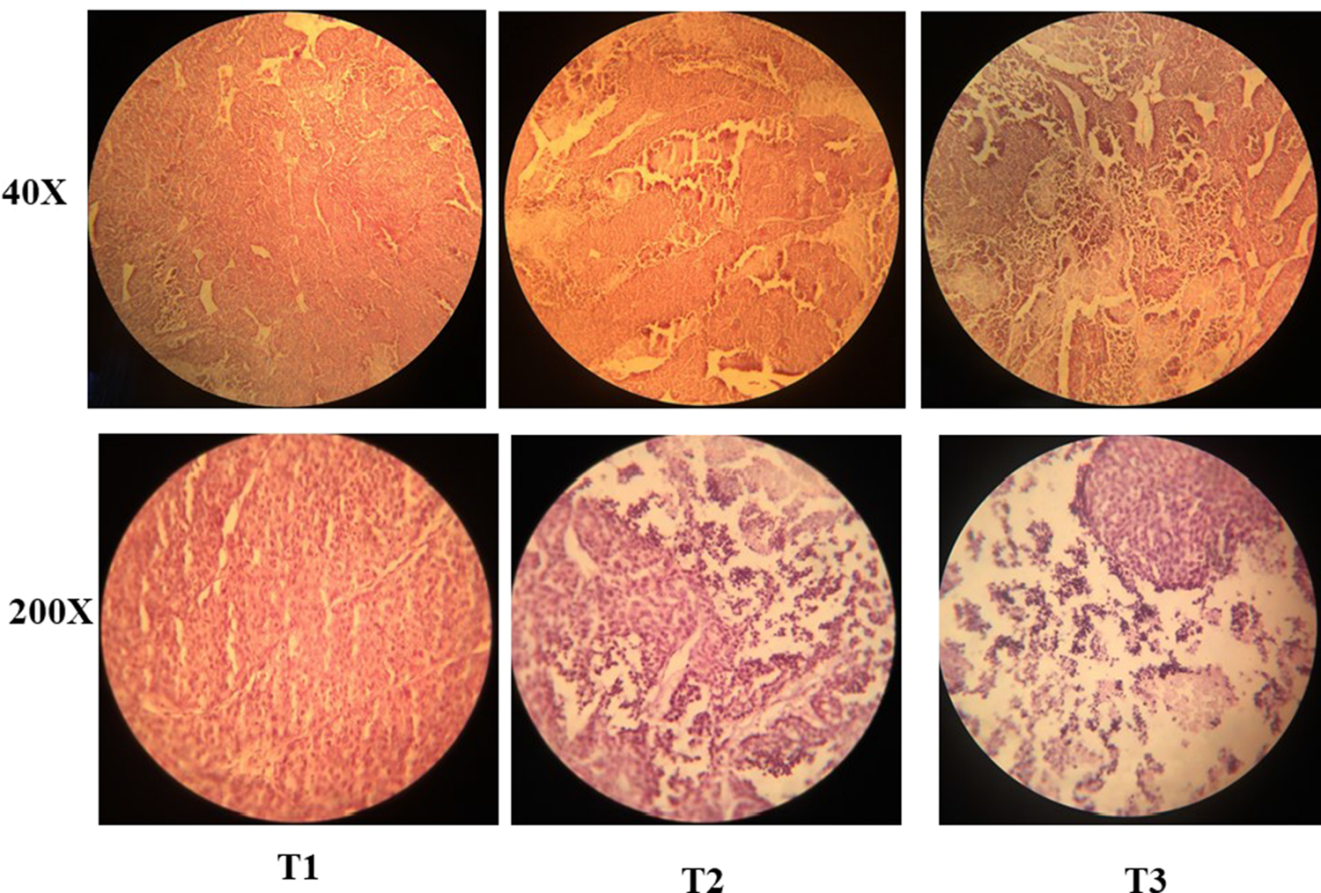
Histopathological analysis of TUBO tumor bearing mice receiving different treatment. T1: TUBO tumor-bearing mice receiving normal food, T2: TUBO tumor-bearing mice receiving 50 mg/kg/BW of Da-PFC-NPs, T3: TUBO tumor-bearing mice receiving 100 mg/kg/BW of Da-PFC-NPs

Figure 7 depicted the histopathological evaluation in TUBO tumor-bearing mice receiving normal food and different dose of Da-PFC-NPs. In the control group (T1), the carcinoma tissue are indicated fully uniform, solid tumor area and without apoptotic cell. On the hand, the mice administrated with different doses of nanoparticles the apoptotic cell were found and cell density decreased. In a study Rahmati et al. [13] demonstrated the anti-tumor effects of α-terpineol-PLGA nanoparticles coated with folic acid-chitosan. They showed that synthesized nanoparticle decreases the tomur size and enhance the apoptotic area in TUBO tumor-bearing mice group.

### Apoptosis-related genes in the TUBO tumor

**Fig. 8:**
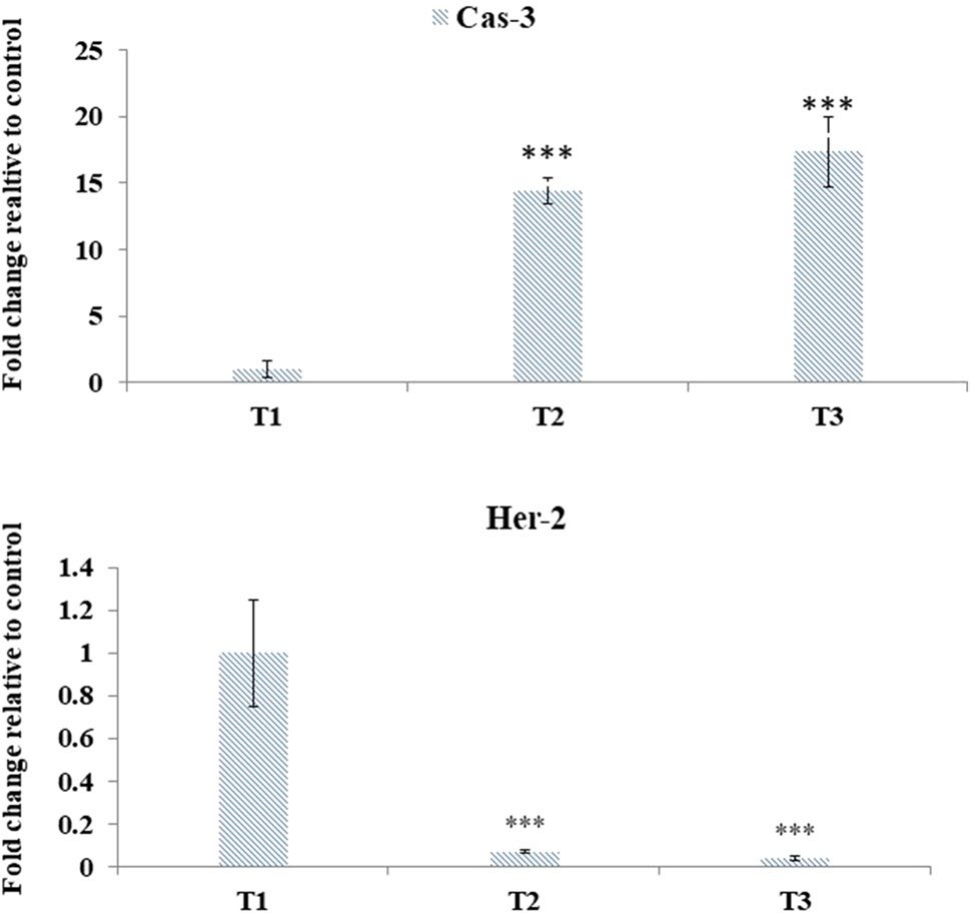
The changes in the expression of Caspase 3 and HER2 genes in the TUBO tumor in the mice receiving different treatments. T1: TUBO tumor-bearing mice receiving normal food, T2: TUBO tumor-bearing mice receiving 50 mg/kg/BW of Da-PFC-NPs, T3: TUBO tumor-bearing mice receiving 100 mg/kg/BW of Da-PFC-NPs. The analyses were performed in triplicates and ***p < 0.001)

The resistance to apoptosis is one of the signs of cancer and the changes in expression of fundamental genes including HER2 and Caspase 3 result in to suppression of apoptosis and inhibit the cell proliferation and tumor growth [20]. The obtained results illustrated that Da-PFC-NPs significantly up-regulated of caspase 3 gene and down-regulated the HER2 gene expression (Fig. 8). Similar to the observation made in this study, Moeini et al. [21] reported the apoptotic activities of nanophytosome‑loaded phenolic compounds from fruit of *Juniperus polycarpos* against breast cancer in mice model. They revealed that nanophytosome encapsulated phenolic compounds notably developed the expression of bax and caspase genes and decrease the bcl2 gene in tumor cells.

## Conclusion

The encapsulation of diosgenin in PLGA nanoparticles was successfully conducted. These nanoparticles were then coated with folic acid-chitosan and characterized, resulting in the best formulation which showed monodispersity, appropriate size and high stability. Evaluation of the nanoparticles' anticancer properties in in vitro and in vivo models showed that Da-PFC-NPs significantly inhibited TUBO breast cancer cell growth in a dose-dependent manner, while also inducing apoptosis in the tumor cells through caspase 3 and HER2 genes. Based on these findings, Da-PFC-NPs could be used as a platform for the delivery of diosgenin and developed into a therapeutic drugs. However, it is important to note that this study did not investigate potential side effects or toxicity. The in vivo model used may also not fully replicate the complexity of human physiology and tumor microenvironments, which may limit the generalizability of the results to human cancer patients. Further studies are needed to address these limitations and explore the long-term efficacy and safety of the Da-PFC-NPs.

## Data Availability

The datasets applied during the current study are available on reasonable request.
